# Predictors of Mortality in Traumatic Intracranial Hemorrhage: A National Trauma Data Bank Study

**DOI:** 10.3389/fneur.2020.587587

**Published:** 2020-11-17

**Authors:** Esther Wu, Siddharth Marthi, Wael F. Asaad

**Affiliations:** ^1^Department of Neurosurgery, Warren Alpert Medical School of Brown University, Providence, RI, United States; ^2^Carney Institute for Brain Science, Brown University, Providence, RI, United States; ^3^Department of Neuroscience, Brown University, Providence, RI, United States; ^4^Norman Prince Neurosciences Institute, Rhode Island Hospital, Providence, RI, United States; ^5^Department of Neurosurgery, Rhode Island Hospital, Providence, RI, United States

**Keywords:** national trauma data bank, support vector machine, traumatic brain injury, traumatic intracranial hemorrhage, mortality predictors

## Abstract

**Background/Objective:** Traumatic intracranial hemorrhage (tICH) accounts for significant trauma morbidity and mortality. Several studies have developed prognostic models for tICH outcomes, but previous models face limitations, including poor generalizability and limited accuracy. The objective was to develop a prognostic model and determine predictors of mortality using the largest trauma database in the U.S., applying rigorous analytical methodology with true hold-out-set model validation.

**Methods:** We identified 248,536 patients in the National Trauma Data Bank (NTDB) from 2012 to 2016 with a diagnosis code associated with tICH. For each admission, we collected demographic information, systolic blood pressure, blood alcohol level (BAL), Glasgow Coma Score (GCS), Injury Severity Score (ISS), presence of epidural/subdural/subarachnoid/intraparenchymal hemorrhage, comorbidities, complications, trauma center level, and trauma center region. Our final study population was 212,666 patients following exclusion of records with missing data. The dependent variable was patient death. Linear support vector machine (SVM) classification was carried out with recursive feature selection. Model performance was assessed using holdout 10-fold cross-validation.

**Results:** Cross-validation demonstrated a mean accuracy of 0.792 (95% CI 0.783–0.799). Accuracy, precision, recall, and AUC were 0.827, 0.309, 0.750, and 0.791, respectively. In the final model, high ISS, advanced age, subdural hemorrhage, and subarachnoid hemorrhage were associated with increased mortality, while high GCS verbal and motor subscores, current smoker, BAL beyond the legal limit, and level 1 trauma center were associated with decreased mortality.

**Conclusions:** A linear SVM model was developed for tICH, with nine features selected as predictors of mortality. These findings are applicable to multiple hemorrhage subtypes and may benefit the triage of high risk patients upon admission. While many studies have attempted to create models to predict mortality in TBI, we sought to confirm those predictors using modern modeling approaches, machine learning, and true hold-out test sets, using the largest available TBI database in the U.S. We find that while the predictors we identify are consistent with prior reports, overall prediction accuracy is somewhat lower than prior reports when assessed more rigorously.

## Introduction

Traumatic brain injury (TBI) is a leading cause of death worldwide, with an annual incidence of ~1.7 million in the United States (1, 2). Among these patients, traumatic intracranial hemorrhages (tICH) are common findings, occurring in up to half of patients and are associated with significant morbidity and mortality (3). Prognostic modeling provides a unique opportunity to aid clinical reasoning and streamline decision making, utilizing patient data to predict various outcomes of TBI.

Several studies have developed prognostic models for traumatic brain injury outcomes using clinical and radiographic data. Such models include age, Glasgow Coma Score (GCS), pupil reactivity, major extracranial injury, time from injury to presentation, hypotensive episode post-injury, motor ability, presence of subarachnoid/subdural hemorrhage, blood alcohol concentration, antiplatelet/anticoagulant use, and Injury Severity Score as predictors of mortality (3–10). However, these models are limited by unknown generalizability to broader populations, limited accuracy, and the large number of variables needed to predict outcomes. In addition, very few have been built upon a complete, national database and utilize a true “hold-out set” for validation. Compared to models which utilize simple cross validation without a hold-out set and have a tendency to show inflated performance as a result of tuning of hyper parameters to the data set, a model built with a true hold-out set is more robust and therefore more generalizable. The primary objective of this study was to determine predictors of mortality using the largest trauma registry in the United States, applying rigorous analytical methodology with true hold-out-set model validation.

## Methods

### Study Population

Following Institutional Review board approval (IRB Registration #: 00000396, 00000482, 00004624), data were retrospectively collected from the National Trauma Data Bank, the largest and essentially all-encompassing aggregation of U.S. trauma registry data. A series of 4,339,668 patients were admitted between January 1, 2012 and December 31, 2016. For years 2012–2015, patients were identified by International Classification of Diseases, Ninth Revision (ICD-9) codes corresponding to specific tICH subtypes: epidural (852.4, 852.5), subdural (852.2, 852.3), subarachnoid (852.0, 852.1), or intraparenchymal hemorrhage (851.0, 851.1, 851.4, 851.5, 851.8, 851.9). For 2016, patients were identified via ICD-10 codes: epidural (S064X), subdural (S065X), subarachnoid (S066X), or intraparenchymal hemorrhage (S0633, S0637, S0638). A total of 248,546 patients from 2012 to 2016 met the criteria for tICH.

Demographic and clinical data were collected for each patient including, sex, age, race, ethnicity, systolic blood pressure, blood alcohol concentration, Glasgow Coma Score (GCS) subscores, Injury Severity Score (ISS), tICH type, comorbidities (CVA, residual neurologic deficit, diabetes, smoker), complications (stroke/CVA), trauma center level, and trauma center region. Patients with missing data in any of the aforementioned fields were removed from the analysis to yield the final study population (*n* = 212,666).

Because this analysis involved only the national database and its de-identified data, we did not need to obtain informed consent from human subjects.

### Statistical Analysis

The dependent variable in the analysis was patient death as defined by in-hospital mortality or discharge to hospice. Discharge to hospice was equated to mortality in this study given that the majority of patients who are discharged to hospice following traumatic brain injury die within 30 days (11). The independent variables studied included sex, age, race, ethnicity, systolic blood pressure, blood alcohol concentration, GCS-Verbal, GCS-Eye, GCS-Motor, ISS, presence of epidural hemorrhage, presence of subdural hemorrhage, presence of subarachnoid hemorrhage, presence of intraparenchymal hemorrhage, comorbidities, complications, trauma center level, and trauma center region. Variables were selected if they were available in the NTDB and if previous literature had either hypothesized or identified an association of the variable with mortality.

Before model development and training, numerical measures were scaled into continuous variables bounded by 0 and 1, and the data distribution was balanced using the synthetic minority oversampling technique (SMOTE). Eighty percentage of the data set was used for initial training and testing, while 20% of the data was used as a final hold-out testing set.

The Python-based (www.python.org) sklearn library implementation of linear SVM uses certain parameters in order to generate the optimal hyperplane: C, dual, and penalty. C, or cost, indicates the size of the margins surrounding the hyperplane, where a larger C will create a hyperplane with smaller margins. Therefore, C is modified to influence the number of data points that are misclassified when training. Dual specifies whether the model will solve the dual or primal optimization problem when run on the training set. Penalty specifies whether L1 or L2 regularization is used when calculating penalty for the model prediction (12).

In order to determine the optimal settings for each of these parameters, a series of SVM models were generated to select the model providing the best predictive performance. In the model creation, we incrementally changed C between 0.0001 and 5 to identify the value which provided the maximal predictive scores. Due to the number of datapoints and relatively low number of features in our dataset, it was preferable to solve the primal optimization problem (13). Further, as L1 regularization is conventionally used to eliminate features as predictive contributors altogether, penalty was set to L1 in order to aid feature selection (14).

Using these parameters, linear support vector machine (SVM) was carried out and trained on a random 80% training set. Initial model performance was assessed using 10-fold cross-validation within this training set. Recursive feature elimination (RFE) was used to consider smaller and smaller subsets of variables in order to identify the most important and optimal number of features without sacrificing accuracy. This new linear SVM with RFE was trained once again on the 80% training set data. Finally, the SVM was tested on the remaining 20% of data, a true hold-out data set. In this way, the generalizability of hyperparameters selected during the initial cross-validation step could be assessed in a rigorous fashion. Model accuracy, precision, and recall were assessed on this hold-out set. A receiver operating characteristic (ROC) curve was generated.

For each data point, the trained linear SVM can use the values of the independent variables to calculate the probability of mortality for each patient. The linear SVM's decision function was calibrated using Platt's method to increase probability accuracy (15), and probabilities for each data point were calculated. The values were used to develop a *post hoc* risk stratification in order to better visualize how risk status is distributed across the study population. Four subgroups were chosen based on tICH mortality risk stratification thresholds used in past literature (4), and based on the calculated probabilities, the study population was split into four subgroups: Grade I (<5% predicted mortality), Grade II (5–15% predicted mortality), Grade III (15–40% predicted mortality), and Grade IV (> 40% predicted mortality). For each subgroup, predicted and actual mortality were compared to assess whether the predictive value of the SVM was maintained within each subgroup.

## Results

### Study Population

**Figure 1:**
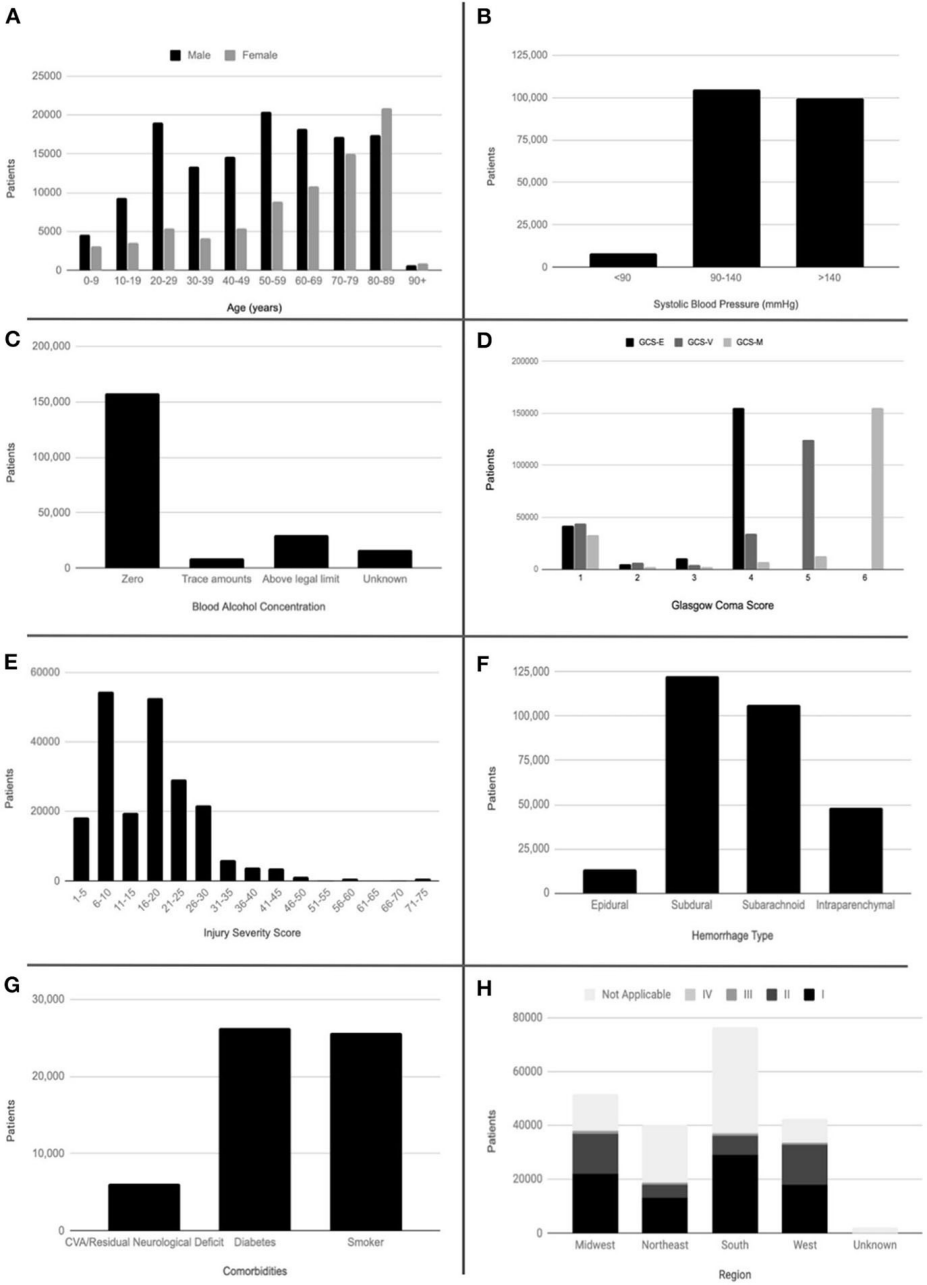
Histograms of demographic and clinical data. **(A)** Patient age by sex. **(B)** Systolic blood pressure. **(C)** Blood alcohol concentration. **(D)** Glasgow Coma Subscores. **(E)** Injury Severity Score. **(F)** Hemorrhage type. **(G)** Comorbidities. **(H)** Trauma center level by region. CVA, cerebral vascular accident; GCS-E, Glasgow Coma Score–Eye; GCS-V, Glasgow Coma Score–Verbal; GCS-M, Glasgow Coma Score–Motor.

77,938 (36.6%) the 212,666 selected patients in the study were female with a mean age at admission of 54.3 years. Of the total studied population, 1,910 patients were self-reported American Indian, 4,912 Asian, 20,334 African American, 550 Native Hawaiian or other Pacific Islander, 160,044 White, and 16,248 other. 19,374 (9.1%) reported Hispanic or Latino ethnicity. The mean systolic blood pressure was 139 mmHg. 29,501 (13.9%) patients had a blood alcohol concentration above the legal limit, 8,560 (4.0%) had a blood alcohol concentration below the legal limit, and 157,854 (74.2%) had a blood alcohol concentration of zero. Mean total GCS was 12.3, while mean ISS was 17.5. 13,156 (6.2%) patients had epidural hemorrhage, 122,772 (57.7%) patients had subdural hemorrhage, 106,359 (50.0%) patients had subarachnoid hemorrhage, and 48,352 (22.7%) patients had intraparenchymal hemorrhage. 62,273 (29.3%) patients had greater than one type of hemorrhage with subdural hemorrhage and subarachnoid hemorrhage being the most common combination. Pre-existing comorbidities evaluated included CVA/residual neurologic deficit, diabetes, and current smoker. 6,118 (2.9%) patients had CVA/residual neurologic deficit, 26,265 (12.4%) had diabetes, and 25,659 (12.1%) were current smokers. Medical complications occurring during the patients' stay included CVA/stroke in 1,485 (0.7%) patients. 82,544 (38.8%) patients were seen at a Level 1 Trauma Center (comprehensive regional resource capable of providing total care for every aspect of injury), 41,335 (19.4%) patients at a Level 2 Trauma Center (able to initiate definitive care for all injured patients), 3,412 (1.6%) patients at a Level 3 Trauma Center (able to provide prompt assessment, resuscitation, surgery, intensive care and stabilization), and 147 (0.1%) patients at a Level 4 Trauma Center (able to provide advanced trauma life support prior to transfer of patients to a higher level trauma center). 51,774 (24.3%) patients were seen at a trauma center in the Midwest, 40,047 (18.8%) in the Northeast, 76,456 (36.0%) in the South, and 42,399 (19.9%) in the West. 19,140 (9.0%) patients had a disposition of death or hospice (Table 1, Figure 1). The mortality rate of the population removed due to missing data was 11.3%. An unpaired samples *t*-test showed this rate was not significantly different from that of the study population (*p* = 0.529).

**Table 1:** Study population demographics.

Sex	
• Male	134,728 (63.4%)
• Female	77,938 (36.6%)
Age (years)	mean = 54.3, standard deviation = 24.2
• 0–9	7,741 (3.6%)
• 10–19	12,891 (6.1%)
• 20–29	24,381 (11.5%)
• 30–39	17,458 (8.2%)
• 40–49	19,983 (9.4%)
• 50–59	29,329 (13.8%)
• 60–69	28,987 (13.6%)
• 70–79	32,150 (15.1%)
• 80–89	38,216 (18.0%)
• 90+	1,531 (0.7%)
Race	
• American Indian	1,910
• Asian	4,912
• African American	20,334
• Native Hawaiian or Other Pacific Islander	550
• White	160,044
• Other Race	16,248
• Unknown	8,779
Ethnicity	
• Hispanic or Latino	19,374 (9.1%)
• Not Hispanic or Latino	161,397 (75.9%)
• Unknown	31,896 (15.0%)
Systolic blood pressure (mmHg)	median = 138, range = 0–300
• <90	8,178 (3.8%)
• 90–140	104,714 (49.2%)
•>140	99,775 (46.9%)
Blood alcohol concentration	
• Zero	157,854 (74.2%)
• Trace amounts	8,560 (4.0%)
• Above legal limit	29,501 (13.9%)
• Unknown	16,752 (7.9%)
Glasgow coma score	median = 15, range = 3–15
• Mild (13–15)	156,654 (73.7%)
• Moderate (9–12)	12,132 (5.7%)
• Severe (3–8)	43,881 (20.6%)
Injury severity score	median = 16, range = 1–75
• Minor trauma (1–15)	92,376 (43.4%)
• Major trauma (16–75)	120,291 (56.6%)
Epidural hemorrhage	
• Present	13,156 (6.2%)
• Absent	199,511 (93.8%)
Subdural hemorrhage	
• Present	122,772 (57.7%)
• Absent	89,895 (42.3%)
Subarachnoid hemorrhage	
• Present	106,359 (50.0%)
• Absent	106.308 (50.0%)
Intraparenchymal hemorrhage	
• Present	48,352 (22.7%)
• Absent	164,315 (77.3%)
Comorbidities	
• CVA/residual neurological deficit	6,118 (2.9%)
• Diabetes	26,265 (12.4%)
• Smoker	25,659 (12.1%)
Complications	
• Stroke/CVA	1,485 (0.7%)
Trauma center level	
• I	82,544 (38.8%)
• II	41,335 (19.4%)
• III	3,412 (1.6%)
• IV	147 (0.1%)
• Not applicable	85,229 (40.1%)
Trauma center region	
• Midwest	51,774 (24.3%)
• Northeast	40,047 (18.8%)
• South	76,456 (36.0%)
• West	42,399 (19.9%)
• Unknown	1,991 (0.9%)
Outcome	
• Alive at discharge	193,527 (91.0%)
• Death/discharge to hospice	19,140 (9.0%)

### Model Evaluation

**Figure 2:**
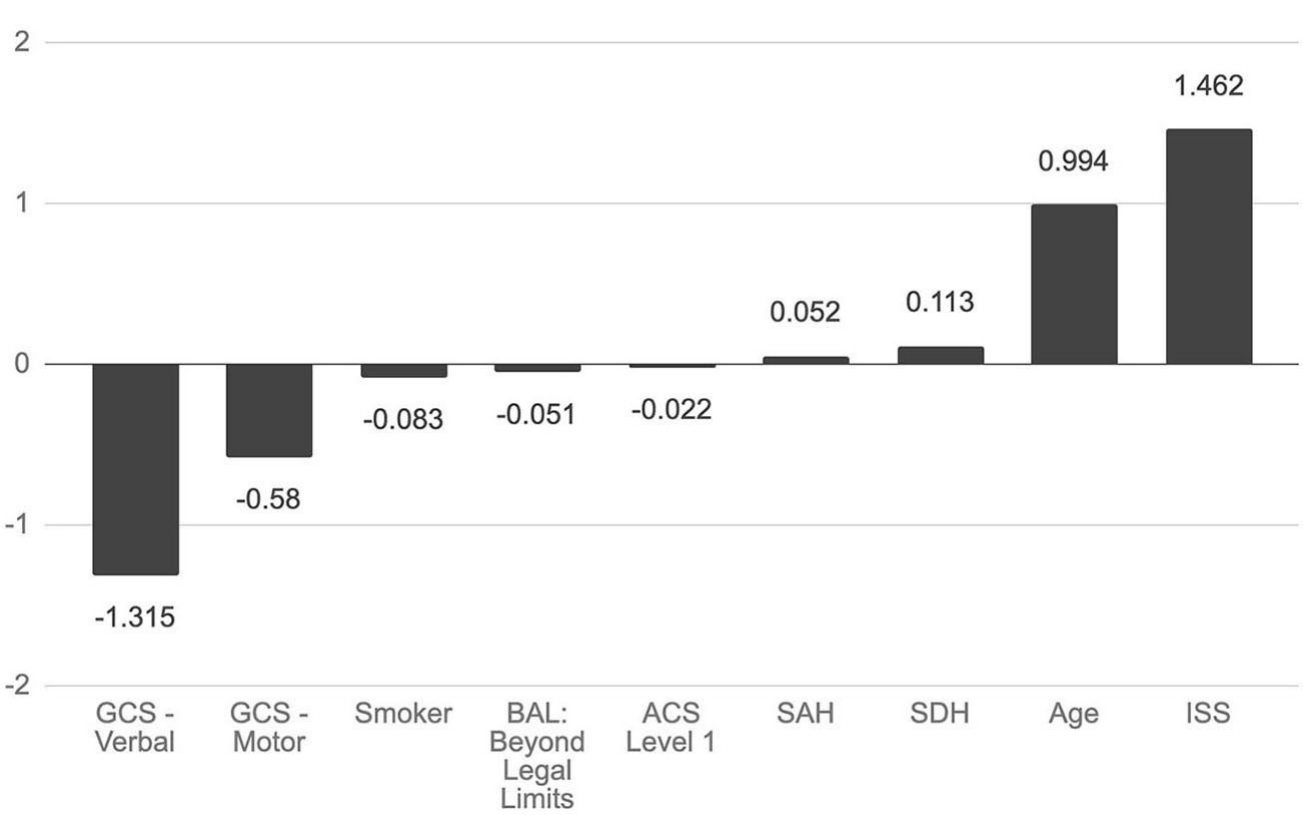
Association of selected features with mortality. Nine features were selected in constructing a hyperplane to separate mortality outcomes. The magnitude of the feature's coefficient is proportional to its importance in predicting mortality outcomes. GCS, Glasgow Coma Score; BAL, blood alcohol level; ACS, American College of Surgeons; SAH, subarachnoid hemorrhage; SDH, subdural hemorrhage; ISS, Injury Severity score.

Cross-validation demonstrated a mean accuracy of 0.792 (95% CI 0.783–0.799). Accuracy for the model, or proportion of correct classifications, was 0.827. Precision, the proportion of true positives to total predicted positives, was 0.309. Recall/sensitivity, the proportion of true positives to total positives, was 0.750. Specificity, the proportion of true negatives to total negatives, was 0.831. Area under the ROC curve (AUC) was 0.791, which describes the model's ability to discriminate between outcomes. In the final model, nine features were selected. High ISS, advanced age, presence of subdural hemorrhage, and presence of subarachnoid hemorrhage were associated with increased mortality, while high GCS-V, high GCS-M, current smoker, blood alcohol level beyond the legal limit, and level 1 trauma center were associated with decreased mortality (Figure 2).

*Post hoc* risk stratification of the study population showed that the 134,161 (63.1%) patients with Grade I tICH (predicted mortality <5%) had an actual total mortality of 1.5% [95% CI: (1.4%, 1.6%)], the 41,899 (19.7%) patients with Grade II tICH (predicted mortality 5–15%) had an actual mortality of 9.8% [95% CI: (9.5%, 10.0%)], the 15,501 (7.3%) patients with Grade III tICH (predicted mortality 15–40%) had an actual mortality of 22.2% [95% CI: (21.5%, 22.7%)], and the 21,105 (9.9%) patients with Grade IV tICH (predicted mortality > 40%) had an actual mortality of 46.6% [95% CI: (45.8%, 47.2%)].

The dataset was trained using several other machine learning algorithms in order to confirm that the use of a linear SVM was appropriate for the characteristics of these data. In comparing the performance of a logistic regression, decision tree classifier, k-nearest neighbors algorithm, Gaussian Naive Bayes classifier, linear discriminant analysis, radial basis function kernel SVM, and polynomial kernel SVM to our linear SVM, performance was highest with the linear SVM model (Table 2). Performance was measured with each model's accuracy of mortality prediction when applied to the testing set.

**Table 2:** Classifier comparison.

**Classifier**	**Accuracy of mortality prediction**
SVM, linear kernel	0.827
SVM, radial basis function kernel	0.791
SVM, polynomial kernel	0.804
Logistic regression	0.801
K-nearest neighbors algorithm	0.810
Decision tree classifier	0.792
Gaussian naïve bayes classifier	0.744
Linear discriminate analysis	0.812

*SVM, Support Vector Machine*.

### *Post-Hoc* Tests

Accounting for variable collinearity is not a priority in support vector machines as it is in other machine learning models, such as regression. In a support vector machine, jointly considering variables, even if they are correlated, improves the predictive power of the model, because the algorithm is not affected by the statistical attributes of the dataset (16). Therefore, the associations that this model identifies are, on their own, not necessarily comparable to those that would be identified by a regression, and cannot be interpreted as such. Therefore, we cannot necessarily conclude that each of the nine features is a unique predictor of mortality.

Previous literature has suggested that SAH is more predictive of TBI mortality when it occurs simultaneously with another tICH, such as SDH (17, 18). We examined the mortality in subgroups of the SAH population, including SAH with concurrent SDH, SAH without SDH, and SAH without other tICH. These groups had mortality rates of 16.67, 6.16, and 5.73%, respectively. Unpaired samples *t*-tests showed that the rates of mortality were significantly different between SAH with concurrent SDH and SAH without SDH (*p* = 0.00821), as well as between SAH with concurrent SDH and SAH without other tICH (*p* = 4.25 × 10^−4^). However, an unpaired samples *t*-test analyzing mortality in the groups of all SAH vs. without SAH produced results that were not statistically significant (*p* = 0.643).

The nine identified variables may not all be obtainable at admission of a patient, decreasing the applicability of the full model in many situations. Therefore, another model was trained only using independent variables that can be easily identified or approximated at admission: presence of epidural hematoma, presence of subdural hematoma, presence of subarachnoid hemorrhage, presence of contusion, age, ISS, GCS-V, GCS-M, and GCS-E. Model evaluation gave an accuracy of 0.806, precision of 0.274, recall of 0.714, and AUC of 0.764. Though performance was somewhat lower than the complete model, a pared-down model such as this may nonetheless be a viable alternative when all nine predictors are not available.

## Discussion

### Model Evaluation

While a variety of models exist, the linear SVM was chosen for its ability to accommodate many independent variables and the limited influence of outliers on model performance. Additionally, given the benefit of the large NTDB data set, a true hold-out set was used to avoid overfitting and allows for a more accurate depiction of model performance. The use of a true hold-out set is a novel approach compared to previously developed models; given the significantly smaller sample sizes seen in comparable studies, the use of a true hold-out set is often not possible. Thus, our model is likely to be more generalizable to broader populations, despite a marginal sacrifice in accuracy and sensitivity (14). Compared to other previously developed models, ours performs comparably well on specificity and AUC, while performing on the lower end for accuracy and sensitivity (Table 3). This discrepancy may have been the result of using a true hold-out set for model validation, which was possible given this study's large patient population.

**Table 3:** Model comparison.

**Paper**	**Accuracy**	**Sensitivity/recall**	**Specificity**	**AUC**	***N***
Current study	82.7	75.0	83.1	0.791	212,666
Powers et al. (4)	88.1	83.0	76.1	n/a	4,100
Rau et al. (10)	97.7	100	97.7	n/a	545
Han et al. (6)	n/a	76.1	82.9	0.87	300
Jacobs et al. (7)	n/a	n/a	n/a	0.86	700
Steyerberg et al. (8)	n/a	n/a	n/a	0.66–0.84	8,509
MRC Crash Trial Collaborators (5)	n/a	n/a	n/a	0.81–0.88	10,008

*AUC, Area under the ROC curve*.

Treatment (e.g., surgery) for each case of tICH was not evaluated as part of the model, given the assumption that patients were treated optimally either medically and/or surgically. Similarly to previously developed models, the goal of this study was not to identify best treatments, but rather, to identify predictors of mortality assuming that patients received the most appropriate care.

### Variable Associations

The associations of higher ISS, advanced age, presence of SDH, presence of SAH, low GCS, level 1 trauma center, and BAL beyond the legal limit with mortality corroborate conclusions made by previous literature (3–10). Prior analyses of the relationship of alcohol consumption and TBI outcomes have produced contradictory conclusions, likely due to differing methods of study and the complex relationship between alcohol intake and the physiologic response to tICH. Though it is hypothesized that low to moderate alcohol intake is protective in TBI due to NMDA receptor and sympathetic nervous system inhibition, high alcohol intake has overwhelmingly been associated with poorer TBI outcomes due to increased cerebral edema and negative effects on neurobehavioral function (19), which this study corroborates. Interestingly, current smoker status was found to be associated with decreased mortality. Smaller past studies have found this factor to be a poor predictor of outcomes in TBI (20), and further study into this variable in particular is therefore warranted. However, one potential explanation for the beneficial effect identified by this study is the neuroprotective effect of nicotine through modulation of the cholinergic anti-inflammatory pathway (21, 22).

Risk stratification categories developed by models such as this one could have clinical utility. For example, for incoming patients with tICH, the nine associated variables could be collected, and the model would use historical national data to estimate a mortality probability that would sort patients into appropriate prognostic groups, thereby assisting with triage. Ongoing data collection via the NTDB can be used to improve the model's performance over time.

One important application of these sorts of models is potentially to guide enrollment in TBI-related studies. Many studies in this field have likely been limited by overly-broad enrollment criteria such that they included patients who would likely have done well or, at the other extreme, would likely have done poorly, regardless of the experimental intervention (23). Such an approach can severely limit a clinical trial's power. Ideally, such studies would focus on the enrollment of patients whose outcomes are less certain (e.g., Grade II or Grade III tICH, as defined here), and are therefore potentially more modifiable. A Grade “calculator” (https://ntdbmortalitycalculator.github.io/) was developed to allow for broader usability and application of the model. The calculator could be utilized to select patients for clinical trial enrollment as well as better risk-stratify individuals.

### Limitations

Compared to other previously developed models, this current model performs on the lower end of accuracy and sensitivity. However, this is likely attributable to the larger, more diverse study population as well as the use of a true hold-out set which avoids the overfitting likely seen in comparable models. Because this is the first study to predict mortality using the NTDB, the generalizability of these results may be greater.

Further, as shown in the *post-hoc* tests, the nine identified features are limited to being predictors when considered in conjunction with each other, so each may not be independently associated with mortality. Subgroup analyses of the nine variables using alternate machine learning methods could identify whether each variable or specific combinations of variables are more associated with mortality. This would help better define the individual relationship between each variable and mortality, and is therefore warranted in future study.

In addition, this model's scope was limited to cases of tICH and thus, its implications on types of non-hemorrhagic TBI may not be clear. We aimed to focus on factors that are accurate, CT-based predictors. The choice was made to exclude diffuse axonal injury (DAI), concussion, traumatic cerebral edema, and diffuse brain injury from the analysis given poor sensitivity based on CT scan, which is how patients are typically initially evaluated, and further evaluation with MRI is uncommon (24–27).

Medical complications and comorbidities evaluated in the model were limited to those most closely linked to tICH. Additional complications and comorbidities included in the NTDB (Supplementary Tables 1, 2) may be worth evaluating and may point to newfound associations with tICH mortality.

Like all large trauma databases, the NTDB suffers from missing and erroneously entered physiologic data, notably for GCS and SBP values, which can result in unexpected data distributions. Though the use of complete case analysis in studies of large trauma databases is standard (28), its use may influence study results. Optimal data imputation methods for the NTDB should be identified and applied to future studies to minimize bias.

Further, because the NTDB aims to provide broad and general data spanning all fields of trauma care, this study is particularly subject to limitations of secondary data analysis. The NTDB does not contain certain clinical factors that would be significant when evaluating tICH prognosis, such as time to treatment and types of interventions. It also does not include known relevant prognostic scores, such as the Marshall CT scan classification score (MCTC). To ensure greater robustness in subsequent studies, national trauma data collection should seek to include additional relevant fields for each data point that are relevant to that field of trauma care.

Lastly, as with other database studies based on ICD codes, errors in coding can contribute to the variability of results.

## Conclusions

tICH plays a critical role in trauma morbidity and mortality. In this study, a linear SVM model to predict mortality was developed and rigorously validated with a true hold-out set for tICH. High ISS, advanced age, presence of subdural hemorrhage, and presence of subarachnoid hemorrhage were associated with increased mortality, while high GCS verbal and motor subscores, current smoker, blood alcohol level beyond the legal limit, and level 1 trauma center were associated with decreased mortality. These findings are applicable to multiple hemorrhage subtypes and can assist in identifying and triaging patients with the highest risk factors for death upon admission. While many studies have attempted to create models to predict mortality in TBI, we sought to confirm those predictors for tICH using modern modeling approaches, machine learning, and true hold-out test sets, using the largest available TBI database in the U.S. We find that while the predictors we identify are consistent with prior reports, overall prediction accuracy is somewhat lower than prior reports when assessed more rigorously.

## Data Availability Statement

Publicly available datasets were analyzed in this study. This data can be found here: National Trauma Data Bank (https://www.facs.org/quality-programs/trauma/tqp/center-programs/ntdb).

## Ethics Statement

Following Institutional Review Board approval (IRB Registration #: 00000396, 00000482, 80 00004624), data were retrospectively collected from the National Trauma Data Bank.

## Author Contributions

EW and SM were equally involved in study design, data acquisition, data analysis, interpretation of data, and manuscript writing. WA was involved in study design, project oversight, and manuscript revision. All authors contributed to the article and approved the submitted version.

## Conflict of Interest

The authors declare that the research was conducted in the absence of any commercial or financial relationships that could be construed as a potential conflict of interest.

## Supplementary Material

Supplementary Figure 1 Histogram of Glasgow Coma Scores (Aggregated Subscores).
