# Supplementary material for: Predictors of Mortality in Traumatic Intracranial Hemorrhage: A National Trauma Data Bank Study
Source: Front Neurol. 2020 Nov 17;11:587587. doi: 10.3389/fneur.2020.587587 (PMC7705094; doi:10.3389/fneur.2020.587587)
Supplement: Supplementary file 1 [file Table_1.docx]

**Table 1**. Hospital Complications included in NTDB

| Other |
| --- |
| *Abdominal compartment syndrome (retired 2011)* |
| *Abdominal fascia left open (retired 2011)* |
| Acute kidney injury |
| Adult respiratory distress syndrome (ARDS) |
| *Base deficit (retired 2011)* |
| *Bleeding (retired 2011)* |
| Cardiac arrest with CPR |
| *Coagulopathy (retired 2011)* |
| *Coma (retired 2011)* |
| Decubitus ulcer |
| Deep surgical site infection |
| Drug or alcohol withdrawal syndrome |
| Deep vein thrombosis (DVT) |
| Extremity compartment syndrome |
| *Graft/prosthesis/flap failure (retired 2016)* |
| *Intracranial pressure (retired 2011)* |
| Myocardial infarction |
| Organ/space surgical site infection |
| *Pneumonia (retired 2016)* |
| Pulmonary embolism |
| Stroke/CVA |
| Superficial surgical site infection |
| *Systemic sepsis (retired 2011)* |
| Unplanned intubation |
| *Wound disruption (retired 2016)* |
| *Urinary tract infection (retired 2016)* |
| *Catheter-related blood stream infection (retired 2016)* |
| Osteomyelitis |
| Unplanned return to the OR |
| Unplanned admission to the ICU |
| Severe sepsis |
| Catheter-associated urinary tract infection (CAUTI) |
| Central line-associated bloodstream infection (CLABSI) |
| Ventilator-associated pneumonia (VAP) |

**Table 2.** Comorbidities included in NTDB

| Other |
| --- |
| Alcohol use disorder |
| *Ascites within 30 days (retired 2015)* |
| Bleeding disorder |
| Currently receiving chemotherapy for cancer |
| Congenital anomalies |
| Congestive heart failure |
| Current smoker |
| Chronic renal failure |
| Cerebrovascular accident (CVA) |
| Diabetes mellitus |
| Disseminated cancer |
| Advanced directive limiting care |
| *Esophageal varices (retired 2015)* |
| Functionally dependent health status |
| History of angina within 30 days |
| History of myocardial infarction |
| History of peripheral vascular disease (PVD) |
| Hypertension requiring medication |
| *Impaired sensorium (retired 2012)* |
| Prematurity |
| *Obesity (retired 2015)* |
| Chronic obstructive pulmonary disease (COPD) |
| Steroid use |
| Cirrhosis |
| Dementia |
| Major psychiatric illness |
| Drug use disorder |
| *Pre-hospital cardiac arrest with resuscitative efforts by healthcare provider (retired 2015)* |
| Attention deficit disorder / attention deficit hyperactivity disorder (ADD/ADHD) |
