# Supplementary figures and images for: Predictors of Mortality in Traumatic Intracranial Hemorrhage: A National Trauma Data Bank Study
Source: Front Neurol. 2020 Nov 17;11:587587. doi: 10.3389/fneur.2020.587587 (PMC7705094; doi:10.3389/fneur.2020.587587)

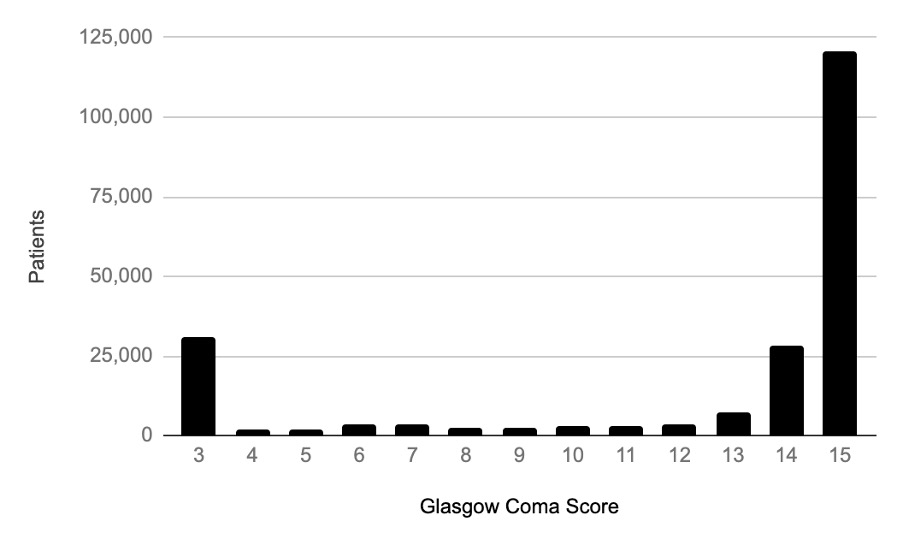

Supplement: Supplementary Figure 1 — Histogram of Glasgow Coma Scores (Aggregated Subscores). [file Image_1.TIFF]
